# Extracellular Vesicles From Kidney Allografts Express miR-218-5p and Alter Th17/Treg Ratios

**DOI:** 10.3389/fimmu.2022.784374

**Published:** 2022-02-23

**Authors:** Alissa K. Rutman, Sarita Negi, Nasim Saberi, Kashif Khan, Jean Tchervenkov, Steven Paraskevas

**Affiliations:** ^1^Department of Surgery, McGill University, Montréal, QC, Canada; ^2^Transplantation Immunology Laboratory, Research Institute of the McGill University Health Centre, Montréal, QC, Canada; ^3^Division of Cardiology and Cardiac Surgery, McGill University Health Centre, Montréal, QC, Canada

**Keywords:** kidney transplantation, extracellular vesicles, delayed graft function (DGF), microRNA, T cell responses

## Abstract

Delayed graft function (DGF) in kidney transplantation is associated with ischemic injury and carries long term functional and immunological risks. Extracellular vesicles (EV) released from allografts may signal a degree of ischemic stress, and are thought to play an important role in the development of anti-donor immunity. Here, we show that kidney perfusate-derived extracellular vesicles (KP-EV) express donor-specific human leukocyte antigen. KP-EV from kidneys that experience DGF increase the T-helper 17 (Th17) to T-regulatory (Treg) ratio in third party peripheral blood mononuclear cells to a greater degree than those from kidneys with immediate function. We report miR-218-5p upregulation in KP-EV of kidney transplant recipients with DGF. Levels of miR-218-5p in KP-EV inversely correlated with recipient eGFR at multiple time points following transplantation. Additionally, the degree of increase in Th17/Treg ratio by KP-EV positively correlated with miR-218-5p expression in KP-EV samples. Taken together, these data provide evidence that KP-EV may contribute to modulating immune responses in transplant recipients. This could lead to novel intervention strategies to inhibit DGF in order to improve graft function and survival.

## Introduction

Kidney transplantation is the preferred treatment for patients with end stage renal disease, improving both quality of life and survival. Some kidneys are more susceptible than others to ischemic injury, which manifests as delayed graft function (DGF), the temporary need for ongoing dialysis after transplantation (1–3). Furthermore, DGF places the recipient at risk of poorer long term outcomes and is associated with higher rates of acute rejection (4–6). While there are known risk factors for DGF, including donor age, there is a poor understanding of the mechanisms that influence the added immunological risk which accompanies the condition. The emergence and advancement of mechanical preservation systems offers the opportunity to study signals released from the kidney which may contribute to priming of the recipient immune system. Recently, several groups have explored donor-derived signals to predict DGF, including *ex vivo* kidney perfusion fluid assessment prior to transplantation and have identified graft-derived risk factors for DGF (7–11).

In the past 10 years, extracellular vesicles (EV) have emerged as important mediators of cellular signaling and as carriers of potent immunomodulatory signals. EV are unique as they package a variety of protein, lipid, RNA and microRNA (miRNA) signals that can be transferred to target cells in a cell-specific manner. Several studies have demonstrated that EV are implicated in innate and adaptive immunity associated with allograft dysfunction (12–14). More recently, EV-containing miRNA have been shown to play important roles in multiple pathologies; accumulating evidence suggests that EV content may vary under specific conditions and disease states (15–18). Additionally, alterations in miRNA and EV-miRNA have been reported in kidney transplant recipients across outcomes (19–22). Recent evidence further suggests that miRNA-containing EV are released by human kidneys under hypothermic machine perfusion and that they may be an important tool to assess graft function in kidney transplantation (23).

In this study, we isolate donor *ex vivo* kidney perfusion fluid extracellular vesicles (KP-EV) and explore their potential immunological role. We document a potential role for miRNA-containing KP-EV in modulating immune responses *in vitro* which may be associated with DGF and poor outcomes in kidney transplant recipients. Our findings suggest that altered miRNA expression in kidney perfusion fluid EV may be associated with DGF and alteration of the balance between Th17 and Treg in kidney transplantation.

## Methods

### Study Approval

The study was approved by the Research Ethics Board of the Research Institute of the McGill University Health Centre (2018-3831) and was conducted in accordance with the principles set out in the declaration of Helsinki. Written informed consent was received from participants before inclusion in the study. Donor samples and patient information were all coded and identified by number.

### Hypothermic Kidney Machine Perfusion and Fluid Collection

Human kidneys were recovered from adult deceased donors and flushed with KPS-1 (Belzer solution, Organ Recovery Systems). Kidneys were placed on the LifePort Kidney Transporter device (Organ Recovery Systems) and perfused with KPS-1 supplemented with mannitol (2.5 g/L) at a systolic pressure of 30 mmHg at 4°C. All kidneys were perfused in 1 L of KPS-1. Samples of perfusion fluid were collected under sterile conditions at the end of perfusion, immediately prior to transplantation, and frozen at -80°C. To reduce selection bias, kidney perfusion fluid samples (from recipients with either DGF or IGF) were selected at random from samples collected and stored between 2017 and 2020 at the McGill University Health Centre Transplant Program.

### Human Subjects and Blood Samples

Peripheral blood samples (10 to 40 mL) were collected from HC (n = 15) in heparin-coated tubes. Peripheral blood mononuclear cells (PBMC) were isolated with Lymphocyte Separation Medium (Wisent). Isolated PBMC were frozen in fetal bovine serum with 10% dimethyl sulfoxide (DMSO) and stored in liquid nitrogen. PBMC were cultured in RPMI 1640 (Gibco) supplemented with 5% human serum (GemCell), 2 mM glutamine (Wisent) and penicillin/streptomycin (100 U/mL penicillin, 100 mg/mL streptomycin; Wisent).

### Extracellular Vesicle Isolation and Labelling

KP-EV were enriched by sequential centrifugation; 40 mL of human kidney perfusion fluid was spun at 1,200 g for 15 minutes to pellet cells and debris. The supernatant was transferred to ultracentrifuge tubes and was spun at 150,000 g for 2 hours. Pellets were washed once and resuspended in 500 µL of PBS. EV samples were frozen at -80°C until further use. For controls, KPS-1 was processed identically and run in parallel.

For KP-EV PBMC interaction experiments, KP-EV or control EV were labeled with 1 µM of CellTracker™ Deep Red (CTDR) (Thermo Fisher) and incubated for 30 minutes at 37°C. KP-EV were washed at 150,000 g for 18 hours, pelleted, resuspended in culture medium and exposed to PBMC (12, 24). Unless otherwise specified, 5 µl of KP-EV or control EV was used in each experiment.

### RNA Isolation

Total RNA was isolated from 10 µl of enriched KP-EV (n=37) using the Qiagen miRNeasy micro kit (Qiagen) as per the manufacturer’s instructions. RNA was frozen at -80°C until further use.

### miRNA Sequencing

Libraries were generated from 1.5 µl of total KP-EV RNA (n=19, 8 IGF, 11 DGF) using the NEBNext Multiplex Small RNA Library Prep Set for Illumina (New England Biolabs), as per the manufacturer’s recommendations. The following modifications were made: 21 PCR cycles were performed as well as a double cleanup. A size selection (between 125 bp and 180 bp) was performed on a Pippin Prep instrument (SAGE Science). Final libraries were quantified using the Kapa Illumina GA with Revised Primers-SYBR Fast Universal kit (Kapa Biosystems). Average size fragment was determined using a LabChip GX (PerkinElmer) instrument. The libraries were normalized and pooled at 3 nM, denatured in 0.05 N NaOH and neutralized using HT1 buffer. ExAMP was added to the mix following the manufacturer’s instructions. The pool (at 200 pM) was loaded on an Illumina cBot and the flowcell was run on a HiSeq 4000 for 2 x 100 cycles (paired-end mode). A phiX library was used as a control and mixed with libraries at 1% level. The Illumina control software HCS HD 3.4.0.38 and the real-time analysis program RTA v. 2.7.7 were used. Bcl2fastq2 v2.20 was then used to demultiplex samples to generate fastq reads.

### miRNA Sequencing Analysis

Reads were trimmed using Trimmomatic v0.36 to remove low quality bases and adapter contamination. Quantification of miRNA features was calculated using mirdeep2 v0.0.8 by mapping to all human miRNA sequences available in miRBase Release 22.1. The mirdeep2 output was collated to produce a table of counts for each of the known miRNA. Read processing was coordinated using custom Nextflow pipeline. Feature counts were normalized to the size of the libraries using edgeR v3.26.8, including removal of miRNA with insufficient abundance and estimation of dispersions. Moderated t-statistics was used to measure differential expression between IGF and DGF using the eBayes function from limma. Identified miRNA for the DGF group were considered differentially expressed if their normalized expression fold changes were ≥1.4 relative to IGF group with unadjusted p-values <0.05. Pathway analyses were performed using Reactome to identify biological pathways of interest. GO analysis, network analysis and visualization were performed using STRING (25, 26).

### Quantitative Real-Time PCR

6.5 µl of KP-EV RNA (n=18, 9 IGF, 9 DGF) was converted to cDNA using the miRCURY LNA Universal RT microRNA PCR kit (Qiagen) according to the manufacturer’s protocol. Prior to cDNA synthesis, synthetic RNA spike-in UniSp6 was added to each sample for normalization. 10 µl of cDNA was diluted 5-fold and quantitative real-time PCR was performed using the miRCURY LNA SYBR Green PCR Kit (Qiagen) using the ViiA 7 real-time PCR system (Applied Biosystems). The expression of miR-218-5p (YP00206034) was normalized to the RNA spike-in UniSp6 (YP00203954). Samples were analyzed in duplicates and differences in miRNA expression were calculated using the ΔΔCt method.

### Electron Microscopy

KP-EV were fixed in 2.5% glutaraldehyde in 0.1M sodium cacodylate buffer. 5 μl were adsorbed to Formvar carbon-coated copper grids and contrasted for whole mount negative staining. Samples were observed using the FEI Tecnai G2 Spirit 120 kV Transmission Electron Microscope. Images were captured on the Advanced Microscopy Techniques XR80C CCD Camera System with AMT Image Capture Engine V601.

### Nanoparticle Tracking Analysis

Kidney perfusion fluid samples were analyzed by the Nanosight NS500 system (Nanosight Ltd) to quantify the mean size and concentration of particles. Kidney perfusion fluid samples were diluted (1:50) in PBS and analyzed with the Nanoparticle Analysis (NTA) System & 1.4 Analytical Software. At least 5 recordings of 30 seconds each were obtained at 37°C with the camera shutter speed set to 30.0 ms, a camera level of 14 and detection threshold set to 9.

### Small Particle Flow Cytometry

25 µl of kidney perfusion fluid samples diluted 1:200 in sterile-filtered PBS (n=10) were incubated with CTDR (1 μM, ThermoFisher Scientific) and anti-CD9 PE (Biolegend), anti-CD63 PE (Biolegend), anti-HLA-DR BV421 (Biolegend), anti-HLA-DQ PE (Biolegend) or anti-HLA-A2 PE (Biolegend) for 30 minutes at room temperature. Small particle flow cytometry was performed using the CytoFLEX system (Beckman Coulter) equipped with 3 lasers (405, 488, and 640 nm wavelength). The 405 nm violet laser for SSC (V-SSC) was selected with 1,800 of manual threshold settings in V-SSC height channel specifically for small particle analysis. Samples were loaded and run with a slow flow rate (10 μl/minute) for 2 minutes until the event rate stabilized; 15 µl of each sample was acquired, with a maximal abort rate of 2.5%. Data were acquired using Cytexpert 2.0 software (Beckman Coulter) and analyzed using Flowjo.

### Flow Cytometry

For interaction and activation assays, PBMC (250,000) were plated in 96-well flat bottom plates and cultured with CTDR labelled EV (or CTDR labelled control EV) for 24 hours at 37°C unless otherwise specified. PBMC were collected and stained for cell surface markers using the following antibodies: anti-CD4 Alexa Fluor 405 (eBioscience), anti-CD8 PE-Cy7 (eBioscience), anti-CD11c PE (Biolegend), anti-CD14 PE-CF594 (BD Bioscience), anti-CD19 Alexa Fluor 488 (eBioscience), anti-CD56 PerCP (eBioscience) and anti-CD69 BV 650 (BD Bioscience).

For T cell assays, culture plates were coated with 1 μg/mL of anti-CD3 mAb (OKT3; eBioscience) for 2 hours. Plates were washed and 250,000 PBMC from healthy controls (HC) (n=4) and 2 μg/mL of anti-CD28 mAb (eBioscience) were co-cultured with KP-EV (n=6-10). At day 7, PBMC were re-stimulated with Cell Stimulation Cocktail (including protein transport inhibitor) (eBioscience) for the final 5 hours of culture and stained for cell surface markers anti-CD4 Alexa Fluor 405 (eBioscience), anti-CD25 BV786 (BD Bioscience). After fixation and permeabilization with the FoxP3 Transcription Factor Fixation/Permeabilization set (eBioscience), PBMC were stained intracellularly for anti-FoxP3 PE-Texas Red (BD Bioscience), anti-IL4 APC (eBioscience), anti-IFNγ APC-Alexa750 (eBioscience) and anti-IL17 PE (eBioscience).

All samples were stained with Fixability Viability Dye eFluor 506 or 780 (eBioscience), to facilitate live-cell gating before cell surface and intracellular staining. Doublets were excluded with forward scatter height against forward scatter area and subsequently side scatter height against side scatter area. Fluorescent minus one controls were used for gating for intracellular cytokine assays. All data were acquired on an LSRFortessa cytometer and analyzed with FlowJo software (Tree Star, Inc.).

### Statistical Analyses

All data are expressed as means +/- SEM. Data were analyzed by two-tailed Student t-test. Correlations were performed with Pearson’s correlation coefficient. Statistical analyses were performed in Prism 7 (GraphPad software Inc.). For all data analyses, p ≤ 0.05 was considered statistically significant.

## Results

### Human Kidneys Release HLA-Expressing EV Under Hypothermic Machine Perfusion

**Figure 1:**
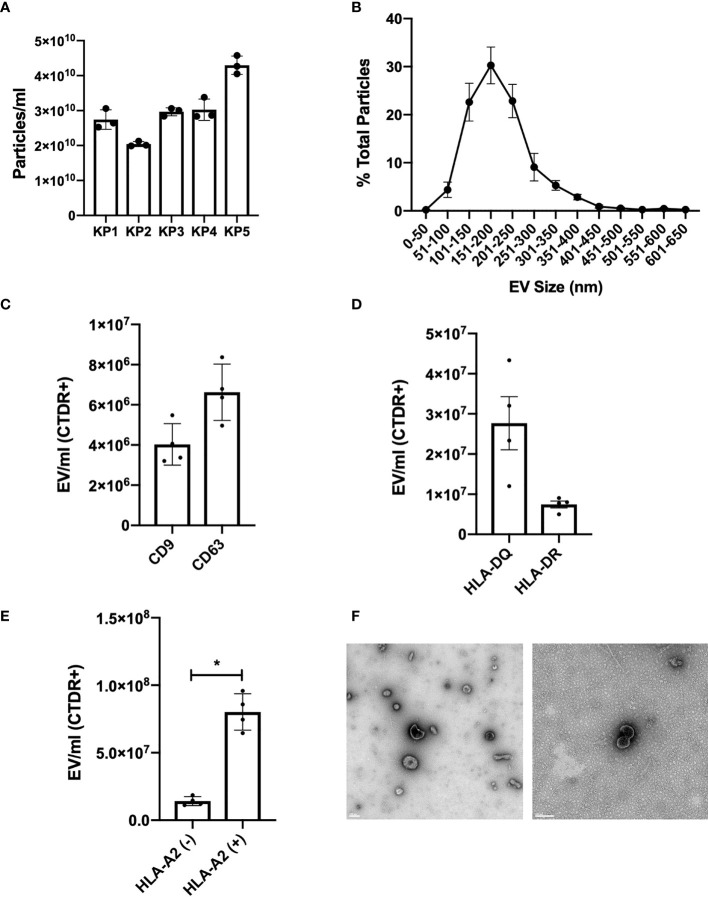
Characterization of KP-EV in human donor kidney perfusion fluid samples. **(A)** Quantification and **(B)** size distribution of kidney perfusion fluid particles by nanoparticle tracking analysis (n=5). **(C)** KP-EV CD9-PE and CD63-PE expression quantification by Cytoflex (n=4). **(D)** KP-EV HLA-DR-BV421 and HLA-DQ-PE expression quantification by Cytoflex (n=4). **(E)** HLA-A2-PE expression of KP-EV derived from HLA-A2 negative donors (n=4) and HLA-A2 positive donors (n=4) by Cytoflex. **(F)** Representative transmission electron microscopy images of KP-EV enriched fractions by whole mount negative staining. Each image represents EV from a different human kidney donor. LEFT: scale bar: 200 nm, magnification: 13 000X. RIGHT: scale bar: 100 nm, magnification: 50 000X. *represents p-values <0.05.

In order to determine whether human kidneys under hypothermic machine perfusion release EV, machine perfusion fluid samples were collected immediately prior to transplantation and analyzed by NTA. NTA revealed a particle size distribution with a peak at 150 nm and a mean number of particles of 2.9 x 10^10^ per ml of perfusion fluid (**Figures 1A, B**). Using small particle flow cytometry *via* Cytoflex, EV were labelled with CTDR and CD9, CD63, HLA-DQ, HLA-DR or HLA-A2. The gating strategy first selected for CTDR+ EV in order to include only membrane bound structures in the analysis; CTDR+ KP-EV were used as negative controls or FMO to gate for positive populations for all markers (**Supplementary 1A, B**). We show that KP-EV express conventional markers CD9 and CD63 (**Figure 1C**). KP-EV were then phenotyped for HLA-specific markers; we show that KP-EV express common tissue HLA markers HLA-DQ and HLA-DR (**Figure 1D**). Next, samples were selected from HLA-A2 positive donors and HLA-A2 negative donors; levels of HLA-A2 were detectable in KP-EV of donors expressing HLA-A2 (**Figure 1E**). KP-EV were then isolated by sequential ultracentrifugation to obtain an EV-enriched fraction; this enriched fraction was used for all experiments going forward. Electron microscopy of these purified KP-EV reveal membrane vesicles in a size range of 100 nm, characteristic of EV (**Figure 1F**). Taken together, these data demonstrate that human kidneys under machine perfusion prior to transplantation release EV expressing common EV markers and donor-specific class I and class II HLA.

### KP-EV Interact With PBMC, Specifically Monocytes and B Cells and Trigger Activation

**Figure 2:**
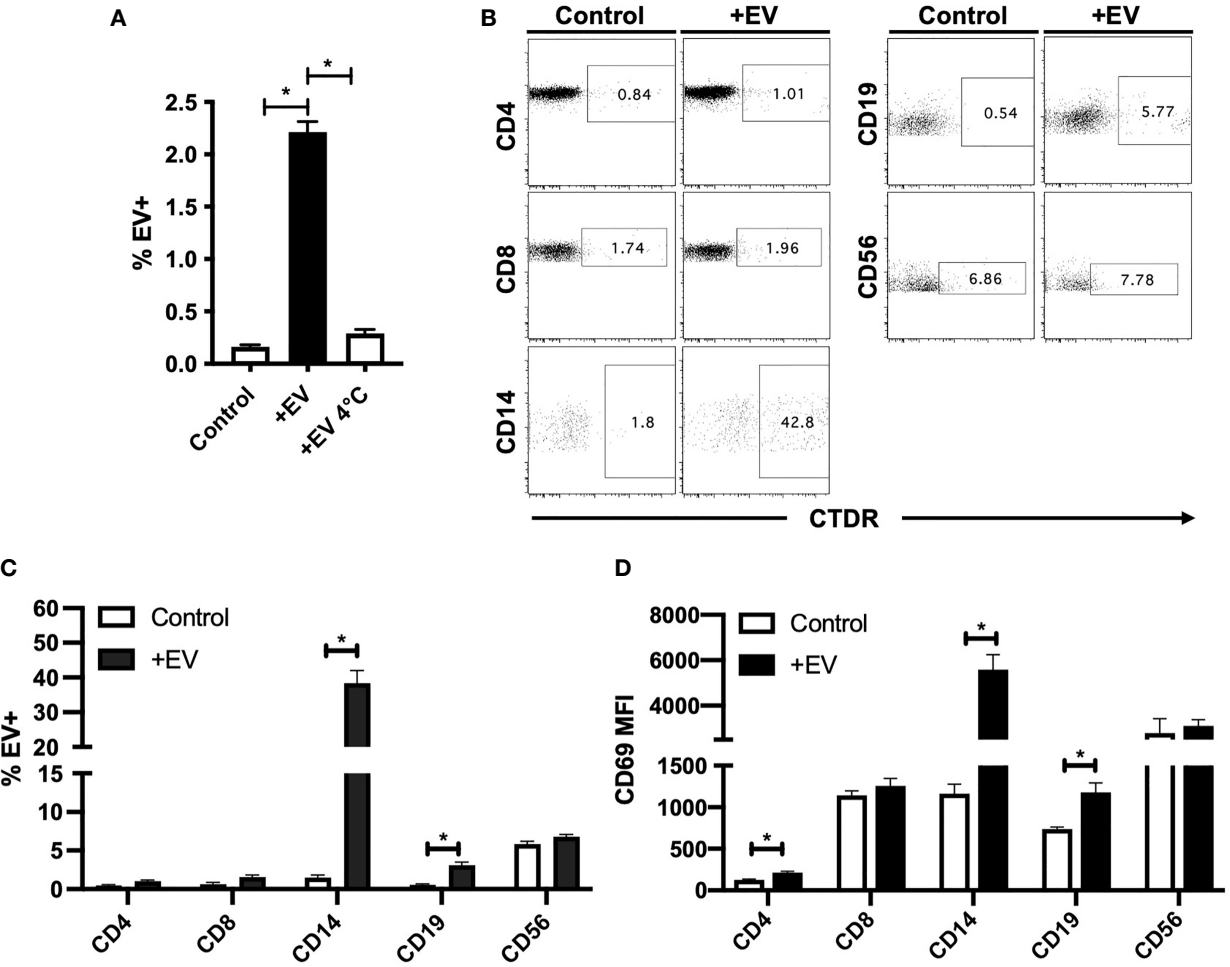
KP-EV interact with PBMC, specifically monocytes and B cells. **(A)** HC PBMC (n=4) were incubated with CTDR (1 μM) labelled KP-EV for 24 hours at either 4°C or 37°C and KP-EV+ PBMC were measured by flow cytometry. **(B, C)** HC PBMC (n=4) were incubated with CTDR (1 μM) labelled KP-EV (n=4) for 24 hours and KP-EV+ PBMC were measured by flow cytometry. As a negative control, KP-EV from KPS-1 were labelled and co-cultured in parallel. Cell surface markers for T cells (CD4^+^ and CD8^+^), B cells (CD19^+^), monocytes (CD14^+^) and NK Cells (CD56^+^) and KP-EV+ cells were analyzed. Representative dot plots show the percentage of cells that are KP-EV+. **(D)** CD69 MFI was measured in gated CD4^+^, CD8^+^, CD14^+^, CD19^+^ and CD56^+^ cells upon PBMC (n=4) exposure to KP-EV (n=4) for 24 hours. *represents p-values ≤0.05.

To investigate the immunological potential of KP-EV, we first co-cultured KP-EV with PBMC from HC. KP-EV were labelled with the fluorescent membrane dye CTDR. KPS-1 preservation solution was stained and processed in parallel as a negative control. We show that upon co-culture with KP-EV, PBMC became EV^+^, as measured by CTDR^+^ cells. This response was reduced at 4°C, suggesting an energy dependent process (**Figure 2A**). To determine which specific cell types in PBMC preparations respond to KP-EV, CTDR labelled KP-EV were cultured with PBMC (24 hours) and analyzed using specific markers for T cells (CD4^+^, CD8^+^), monocytes (CD14^+^), B cells (CD19^+^) and NK cells (CD56^+^). CD14^+^ monocytes were found to be EV+, CD19^+^ B cells to a lesser extent, and no significant changes were observed in other cell types (CD4, CD8, CD56) (**Figures 2B, C**). These data suggest that HC PBMC interact directly with specific antigen presenting cells (APCs). The activation of PBMC in response to KP-EV was then investigated as measured by CD69 mean fluorescence intensity (MFI), an activation marker for lymphocytes and monocytes (27–30). Upon exposure to KP-EV, we detected an increase in CD69 MFI in CD4^+^ T cells, CD14^+^ monocytes and in CD19^+^ B cells, consistent with their activation (**Figure 2D**). These data demonstrate that KP-EV are taken up by CD14^+^ and CD19^+^ cells and lead to the activation of not only CD14^+^ monocytes and CD19^+^ B cells but also CD4^+^ T cells.

### KP-EV Suppress the Induction of Treg and Increase Th17/Treg Ratio, to a Greater Extent With KP-EV of Recipients With DGF

**Figure 3:**
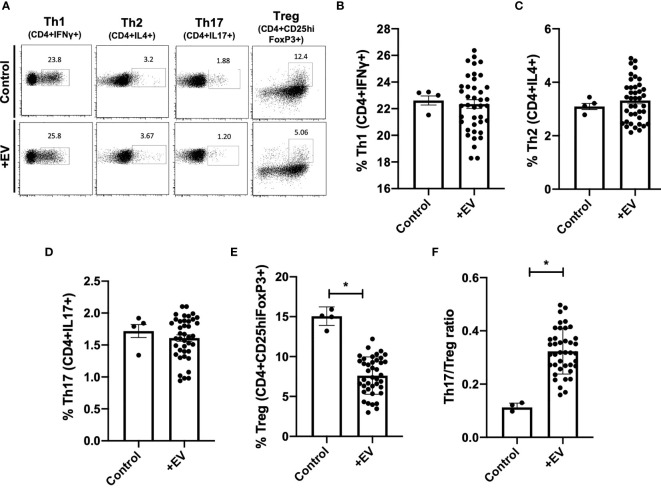
KP-EV increase Th17/Treg ratios in PBMCs. **(A–F)** For T cell activation assays, PBMC were stimulated with 1 μg/mL of anti-CD3 mAb (OKT3) and 2 μg/mL of anti-CD28 mAb; PBMC from HC (n=4) were seeded with KP-EV (n=10). On day 7, cells were stained for CD4, CD25, IFNγ, IL4, IL17 and FoxP3. **(A)** Representative flow cytometry plots and percentages of subpopulations of **(B)** Th1 (CD4^+^IFNγ ^+^), **(C)** Th2 (CD4^+^IL4^+^), **(D)** Th17 (CD4^+^IL17^+^), **(E)** Treg (CD4^+^CD25^hi^FoxP3^+^) and **(F)** the ratio of Th17 to Treg. Each data point represents a KP-EV (n=10) or control EV exposed to HC PBMC (n=4) (technical duplicates). *represents p-values ≤0.05.

**Figure 4:**
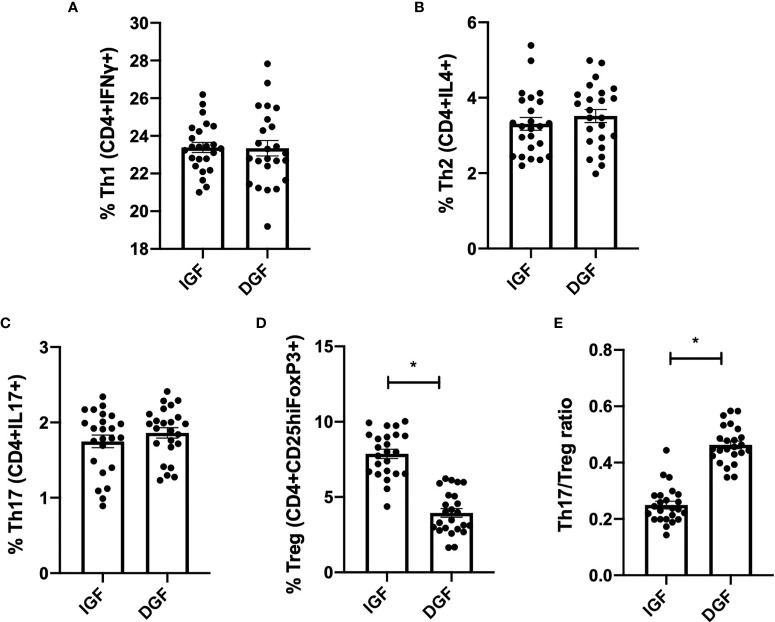
KP-EV suppress the induction of Treg and increase Th17/Treg ratio, to a greater extent in patients with DGF. **(A–E)** For T cell assays, culture plates were coated with 1 μg/mL of anti-CD3 mAb (OKT3) and 2 μg/mL of anti-CD28 mAb; PBMC from HC (n=5) were seeded with KP-EV derived from recipients with IGF (n=6) or DGF (n=6). At day 7, cells were stained for CD4, CD25, IFNγ, IL4, IL17 and FoxP3 and percentages of subpopulations of **(A)** Th1 (CD4+IFNγ+), **(B)** Th2 (CD4+IL4+), **(C)** Th17 (CD4+IL17+), **(D)** Treg (CD4+CD25hiFoxP3+) and **(E)** the ratio of Th17 to Treg were measured by flow cytometry. Each data point represents a KP-EV (n=6 IGF, n=6 DGF) exposed to HC PBMC (n=4) (technical duplicates). *represents p-values ≤0.05.

Next, in order to further examine the effect of KP-EV on T cells, PBMC from HC were treated with anti-CD3 and anti-CD28 and stimulated with KP-EV; on day 7, cells were collected and stained with cell surface markers CD4 and CD25 and intracellular FoxP3, IFNγ, IL4 and IL17 to assess T cell responses to KP-EV. No changes were detectable in CD4^+^IFNγ^+^ (referred to as Th1), CD4^+^IL4^+^ (referred to as Th2) and CD4^+^IL17^+^ (referred to as Th17) cell percentages upon exposure to KP-EV (**Figures 3A–D**). However, following culture with KP-EV, percentage of CD4^+^CD25^hi^FoxP3^+^ cells (referred to as Treg) was significantly reduced (**Figure 3E**). As a result, the Th17 to Treg ratio was increased by approximately 2-fold (**Figure 3F**). Next, we evaluated whether KP-EV derived from donors of recipients with IGF and DGF could differentially downregulate Treg induction and Th17/Treg ratios. Interestingly, KP-EV derived from the donor kidneys of recipients who suffered from DGF downregulated Treg to a greater extent than KP-EV from donor kidneys of recipients with IGF (**Figure 4D**), with no changes in Th1, Th2 or Th17 percentages (**Figures 4A–C**). In addition, the Th17/Treg ratio increased in these cultures (**Figure 4E**). Taken together, these data reveal that kidneys which suffer from DGF release KP-EV which suppress the induction of Treg and increase the Th17/Treg ratio to a greater extent than KP-EV associated with IGF. These results suggest that KP-EV may have inherent features that modulate immune cell responses *in vitro* independent of recipient PBMC characteristics.

### miR-218-5p Expression Is Increased in KP-EV of Recipients With DGF

**Figure 5:**
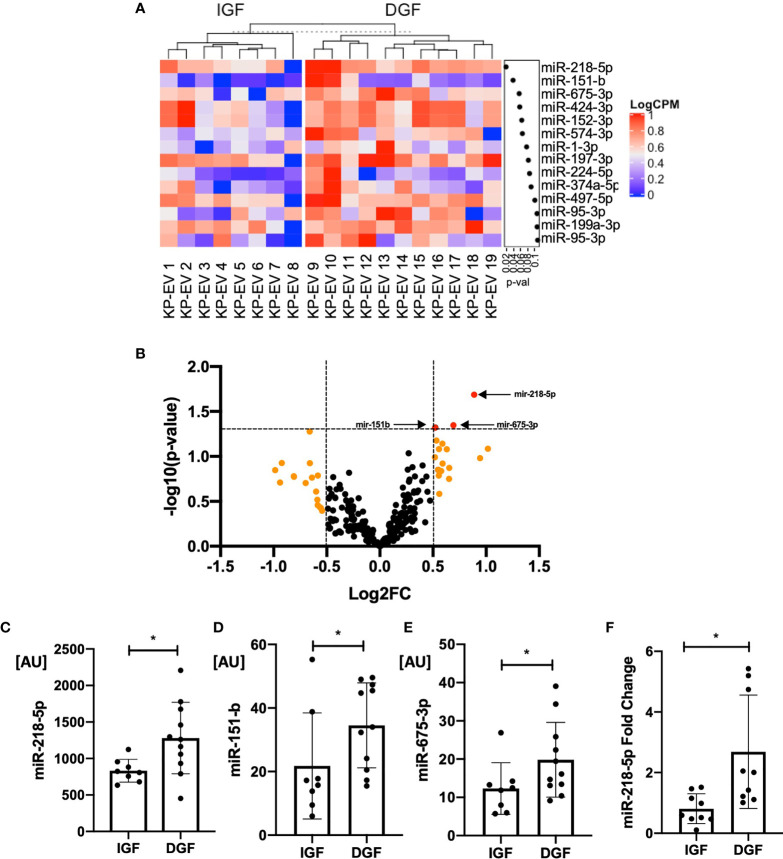
KP-EV miRNA profile differentiates kidney transplant recipients with IGF and DGF. **(A)** Heat map of miRNA expression profile of donor KP-EV of recipients with DGF (n=11) as compared to IGF (n=8). RNA was extracted from KP-EV, which were enriched from the perfusion fluid of human kidney deceased donors. **(B)** Volcano plot of miRNA expression of KP-EV of kidney recipients with DGF as compared to IGF. The fold change of each miRNA is plotted on the X axis in log2 scale and their significance level (–log10-p-value) is plotted on the Y axis. Orange dots represent miRNA with a fold change ≥1.4 and red dots represent miRNA with *p* values ≤0.05. Three miRNA pointed by arrows (red dots) were identified as candidate miRNA upregulated in patients with DGF with a fold change ≥1.4 and *p* values ≤0.05. **(C–E)** Bar graphs of expression values of candidate miRNA levels of the top three hits that were found to be upregulated in recipients with DGF. **(F)** qPCR quantification of miR-218-5p expression in a different set of KP-EV samples of patients with IGF (n=9) and DGF (n=9). *represents p-values ≤0.05.

**Figure 6:**
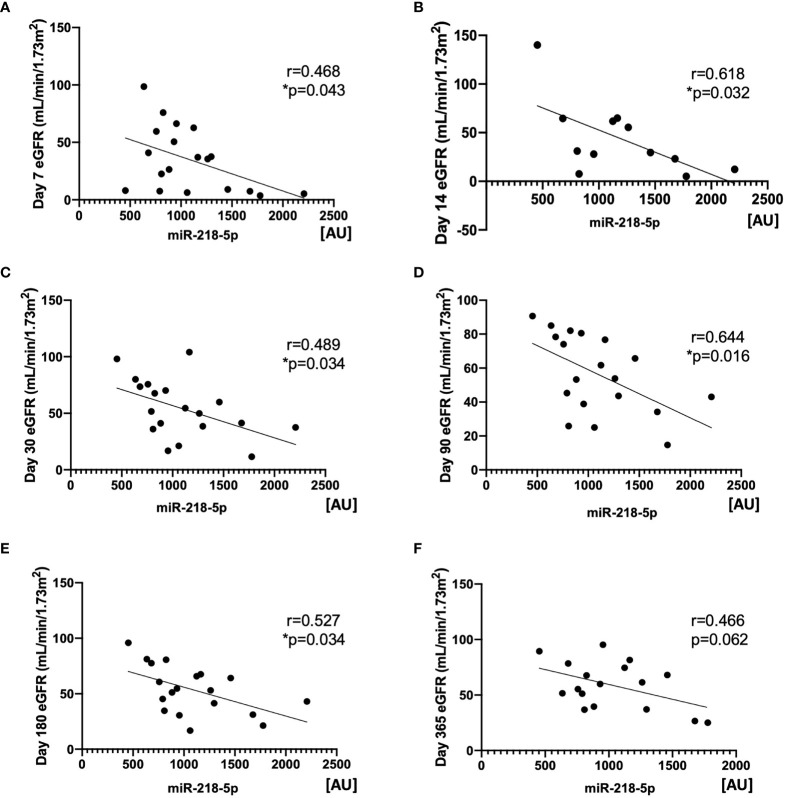
Expression of miR-218-5p in KP-EV negatively correlates with kidney transplant recipient eGFR. **(A-F)** Correlation of KP-EV miR-218-5p expression levels as measured by miRNA sequencing with respective recipient eGFR at **(A)** day 7 **(B)** day 14 **(C)** day 30 **(D)** day 90 **(E)** day 180 and **(F)** day 360 following transplantation. *represents p-values ≤0.05.

EV are known to transport miRNA cargo, in particular, which may account for varying responses in the function of target cells. We next examined potential differences in the miRNA content of KP-EV from kidneys with IGF and DGF which could be associated with these downstream functional changes. No significant differences were found in donor and recipient demographic characteristics in the IGF and DGF groups (**Table 1**). RNA was isolated from 19 KP-EV samples (8 IGF, 11 DGF) and was subjected to miRNA sequencing. Due to low RNA yields, typical for EV preparations, 21 PCR cycles were performed as well as a double cleanup followed by size selection. The average number of reads was 11.9 million. A differential expression analysis of the miRNA was performed between KP-EV of recipients with IGF and DGF. We identified miRNA upregulated in KP-EV of recipients with DGF meeting the criteria of a fold change ≥1.4 and a p-value ≤0.05 (**Figures 5A, B**). miRNA sequencing analyses revealed 3 miRNA that were elevated in KP-EV of recipients with DGF as compared to IGF; miR-218-5p, miR-151-b and miR-675-3p (**Figures 5C–E**). We then evaluated whether a relationship may exist between the expression levels of these miRNA and recipient eGFR following transplantation. KP-EV miR-218-5p expression levels inversely correlated with respective recipient eGFR at day 7, 14, 30, 90 and 180 following transplantation, indicating that elevated miR-218-5p could be associated with DGF and poorer transplant outcomes (**Figures 6A–F**). Potential associations between recipient eGFR and miR-151-b or miR-675-3p were also examined, however, the only significant correlation detectable was between mir-151-b and day 7 eGFR (**Supplementary Figures 2A–F**, **3A–F**). Furthermore, no correlations were found with respect to parameters such as cold ischemic time, pump time and donor eGFR (**Supplementary Figures 4A–C**). However, a positive correlation was found between miR-218-5p and donor age (**Supplementary Figure 4D**). qPCR was then performed with a different set of 18 donors which validated miR-218-5p upregulation in KP-EV of kidney recipients with DGF (**Figure 5F**). Taken together, these data suggest that miR-218-5p is elevated in donor KP-EV of recipients with DGF.

**Table 1:** Donor and recipient characteristics in IGF and DGF cohorts.

Parameter	IGF (n=8)	DGF (n=11)	P value
**Donor**			
Age (yr)	47.2	54.6	0.22
Sex (% male)	62.5 (n=5)	82 (n=9)	0.37
BMI (kg/m^2^)	28.8	28.55	0.47
Creatinine (μmol/L)	71.1	76.4	0.35
eGFR (mL/min/1.73m^2^)	101.1	93.2	0.24
KDRI	1.1	1.3	0.27
CIT (hr)	15.3	17.3	0.13
Pump time (hr)	9.5	7.9	0.28
Recipient*			
Age	53.3	55.6	0.38
Sex (% male)	63 (n=5)	73 (n=8)	0.19
BMI (kg/m^2^)	24.9	28.7	0.08
Transplant #	1.2	1.1	0.41
Dialysis type (% HD)	75 (n=6)	91 (n=10)	0.08
Creatinine (μmol/L)	587.2	735.5	0.16
eGFR (mL/min/1.73m^2^)	10.9	9.5	0.11

*Pre transplant values.

IGF, immediate graft function; DGF, delayed graft function; BMI, body mass index; eGFR, estimated glomerular filtration rate; KDRI, kidney donor risk index; CIT, cold ischemic time; HD, hemodialysis.

### Enriched Biological Processes and Molecular Functions of miR-218-5p Involved in Immune Activation

**Figure 7:**
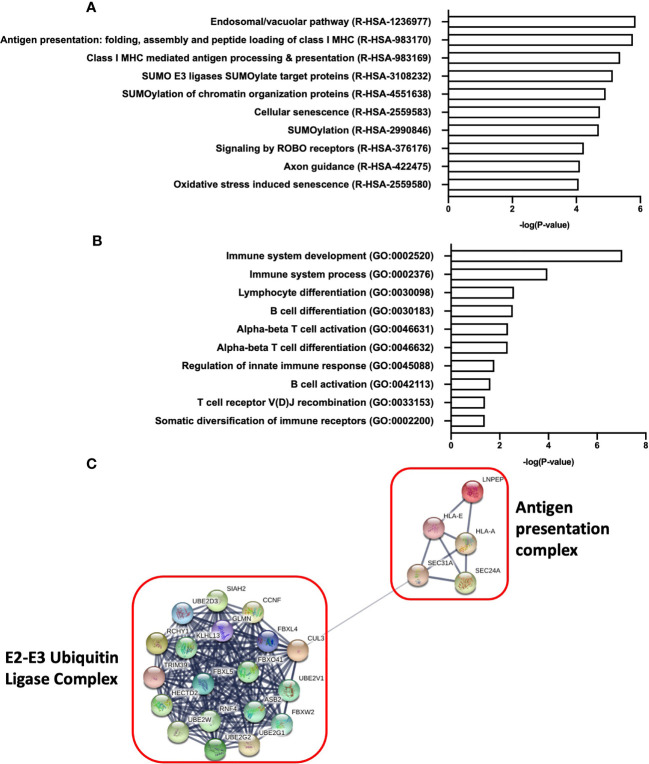
Pathway analysis of miR-218-5p target genes reveal enrichment of pathways involved in immune development and T cell activation. **(A)** Reactome pathway analysis of top 10 of enriched pathways from miR-218-5p target genes. **(B)** Biological processes (Gene Ontology) analysis of pathways involved in immune system development and regulation from miR-218-5p target genes. **(C)** Network analysis of proteins involved in Class I MHC antigen presentation and processing as determined by Reactome analysis. An adjusted p-value ≤0.05 was used as a threshold to select significant terms and pathways.

To uncover the functions and mechanisms of miR-218-5p, pathway enrichment analysis was performed to explore the relationship between the specific gene targets. A total of 900 genes were predictably targeted by miR-218-5p. Reactome pathway analysis of miR-218-5p target genes revealed enrichment of pathways involved in Class I MHC antigen presentation and processing (R-HAS-983170 and R-HAS-983169, **Figure 7A**). GO analysis revealed enrichment of several pathways involved in immune system development and regulation, as well as T and B cell activation (**Figure 7B**). Furthermore, network analysis of the genes involved in Class I MHC mediated antigen processing and presentation (R-HSA-983169) were found to belong to the E3 ubiquitin ligase and E2 ubiquitin conjugating system (**Figure 7C**), which play a role in antigen presentation and T cell activation. Lastly, several of the miR-218-5p target genes identified were critical regulators of T-cell activation such as transcription factors Forkhead Box P1 (FOXP1) and Runt-related transcription factor 2 (RUNX2). These data demonstrate that miR-218-5p may act to regulate T cells responses through several mechanisms including antigen presentation and immune cell activation.

### Degree of Upregulation of Th17/Treg by KP-EV Correlates With KP-EV miR-218-5p Expression

**Figure 8:**
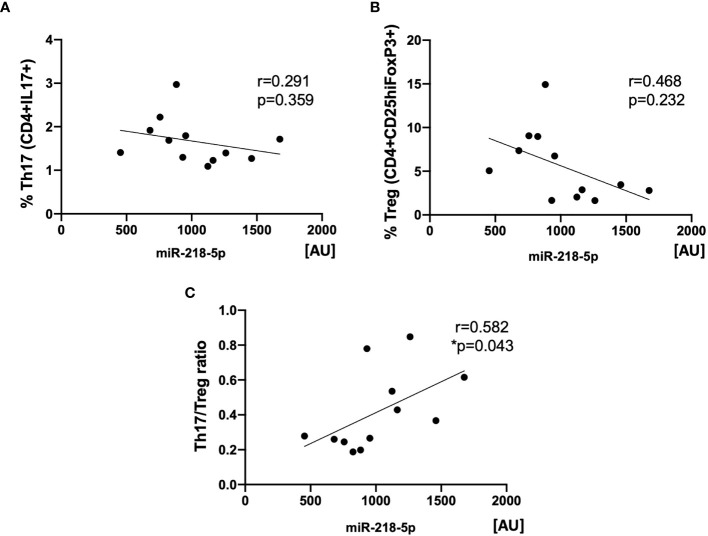
Expression of miR-218-5p in KP-EV positively correlates with Th17/Treg ratios. **(A)** Correlation analysis between miR-218-5p expression in KP-EV and the percentage of Th17 (CD4^+^IL17^+^) upon induction of PBMC with respective KP-EV (n=12). **(B)** Correlation analysis between miR-218-5p expression in KP-EV and the percentage of Treg (CD4^+^CD25^hi^FoxP3^+^) upon induction of PBMC with respective KP-EV. **(C)** Correlation analysis between miR-218-5p expression in KP-EV and Treg/Th17 upon induction of PBMC with respective KP-EV. *represents p-values ≤0.05.

Next, we evaluated whether a relationship may exist between miR-218-5p expression in KP-EV and the induction of Th17 and Treg, as well as their ratio, following stimulation with respective KP-EV. The expression of miR-218-5p in KP-EV samples positively correlated with Th17/Treg ratio. No significant correlation was observed in Th17 or Treg cell frequency alone (**Figures 8A–C**). These finding suggest that miR-218-5p KP-EV expression may be associated with phenotypic profiles favoring a proinflammatory environment in transplant recipients with DGF.

## Discussion

To understand the role of donor KP-EV in kidney transplantation and in modulating immune responses in the context of DGF, we phenotyped EV released by human donor kidneys under hypothermic machine preservation, examined their effects on primary human PBMC and performed miRNA sequencing on their cargo. We provide evidence of donor HLA expression on KP-EV and show that preparations of EV from kidneys which suffered from DGF downregulate Treg induction and upregulate Th17/Treg ratios, potentially favoring a proinflammatory environment. We demonstrate miR-218-5p upregulation in KP-EV of kidney transplant recipients with DGF and show that miR-218-5p expression levels inversely correlated with recipient eGFR. We further show that miR-218-5p expression in KP-EV are associated with their ability to increase Th17/Treg ratio in third party PBMC *in vitro*. To our knowledge, this is the first study to demonstrate an association between KP-EV miRNA, Th17/Treg imbalance and DGF in kidney transplantation.

DGF is a common manifestation of ischemia-reperfusion injury in kidney transplantation, where ongoing dialysis is temporarily required. Although it eventually resolves, it has long lasting consequences to the outcomes of the transplant. DGF has been associated with higher rates of acute cellular rejection and shorter graft survivals (2, 3, 6, 31). Both humoral and cellular immune processes have been shown to play essential roles in allorecognition and graft injury. Allorecognition occurs through two distinct pathways: the “direct” pathway whereby recipient T cells recognize intact donor HLA on the surface of donor APCs and the “indirect” pathway where recipient T cells recognize processed donor HLA-peptides by self-HLA molecules (32–34). Recently, the concept of “semi direct” antigen presentation has emerged in the context of EV; allograft-derived EV can interact with recipient APCs, which then present donor HLA molecules on their surface, a phenomenon known as HLA “cross-dressing” (14, 35–39).

In this study, we show that KP-EV are released by human kidneys under machine perfusion and express EV-specific markers CD9 and CD63 as well as both HLA class I and class II antigens. Other groups have similarly demonstrated that allografts release EV that carry donor HLA to the recipient’s lymphoid organs to trigger alloimmune response (14, 37, 40–42). Gunasekaran et al. showed expression of donor HLA and lung associated self-antigens on EV from serum and bronchoalveolar lavage fluid of lung transplant recipients with acute rejections but not in recipients with stable transplant (43, 44). Few studies in various transplant models have shown that transfer of donor HLA to recipient APCs *via* EV is involved in the perpetuation of alloresponses by T cells leading to graft dysfunction (14, 37, 45).

Knowing that KP-EV express donor-specific HLA and may contribute to modulating immune responses, we examined the functional role of KP-EV on primary human PBMC. Our findings suggest that KP-EV interact directly with CD14^+^ monocytes and CD19^+^ B cells. Uptake of EV from other sources, by these cell types, has been shown by other groups and is an intuitive finding as both monocytes and B cells act in the innate immune response as APCs upon antigen exposure (24, 46–48). Although CD4^+^ T cells did interact directly with KP-EV, we found that they were activated in response to EV exposure, as were CD14^+^ and CD19^+^ cells, as measured by CD69 expression. Previous studies have shown that professional APCs readily acquire and present EV antigens or proteins, leading to downstream T cell stimulation (49–51).

Next, we explored the effect of KP-EV on T cell subsets in PBMC. We show that *in vitro* stimulus with KP-EV reduces the frequency of Treg and increased the ratio of Th17 to Treg. Notably, these responses were greater with KP-EV of recipients with DGF as compared to IGF. The Th17/Treg balance is indispensable for homeostatic immune responses; Treg and Th17‐mediated cellular immune response are important mechanisms accounting for graft failure following renal transplantation (52). Decreased ratio of Th17/Treg can induce immune tolerance and prolong allograft survival whereas elevation of Th17/Treg ratio can lead to allograft rejection (53–57). Elevated Treg are associated with allograft tolerance whereas lower levels are associated with DGF and rejection (58–64); higher levels of Th17 are associated with graft dysfunction and lower levels are protective (65–70). In several other pathological conditions, EV have been shown to have similar effects on Th17 and Treg (71, 72).

It is well documented that EV carry defined cargo which reflect the physiological and pathological features of the organ or tissue of origin. EV represent an efficient and targeted method to exchange specific signals between cells (15, 73). Several groups have identified different miRNA in kidney perfusion fluid that were associated with DGF (20, 74, 75). We demonstrate upregulation of 3 miRNA in KP-EV of grafts that ultimately experience DGF: miR-218-5p, miR-151-b and miR-675-3p. Of the three top candidates, miR-218-5p expression level was found to be the highest and was the sole miRNA to correlate with recipient eGFR at day 7, 14, 30, 90 and 180 following transplantation. Several studies have reported aberrant miR-218 expression under ischemic conditions; miR-218 is highly expressed in renal cells under inflammatory conditions, and inhibition of miR-218 alleviates renal injury (76–78). Overexpression of miR-218 has been shown to trigger apoptosis and pro-inflammatory cytokine production by renal tubule cells (79). In human renal arteries, ischemic injury has been associated with miR-218 upregulation, with increasing levels of miR-218 seen with longer periods of hypoxia (80), identifying miR-218 as pro-inflammatory. Furthermore, through pathway enrichment analysis, we show that miR-218-5p regulates several pathways involved in antigen presentation and immune system regulation, further suggesting its involvement in allorecognition.

We then examined whether a relationship may exist between KP-EV miR-218-5p expression levels and the ability of KP-EV to exert an effect *in vitro* on Treg and Th17. Recent studies have revealed critical functions of several miRNA in influencing differentiation and function of T cells, promoting or suppressing certain T cell subtypes, with several studies identifying correlations between T cell phenotypes and specific miRNA expression (81–88). Similar to our findings, several other studies have shown that EV-miRNA can have an effect on T cells and specifically modify Th17/Treg ratios (72, 89–96). We point towards a functional role of KP-EV in increasing Th17/Treg ratios *in vitro*, however, the underlying mechanisms and relationship between miR-218-5p and regulation of Th17/Treg ratios remains to be explored.

Several limitations in our study hinder our understanding of the true immunomodulatory potential of kidney derived EV in the pathogenesis of DGF. A larger sample size would certainly refine the analysis of the precise effects associated with miR-218-5p expression, as well as T cell responses. Although our results show an association between miR-218-5p expression and the ability of KP-EV to increase Th17/Treg ratios, the association between the two remains unknown. Further *in vitro* and *in vivo* testing must be conducted to establish this. Finally, although our results focused on Th17 and Treg, it is plausible that KP-EV exert an effect on other immune cell types, whether by miR-218-5p or as yet uncharacterized EV cargo. A precise and well understood link between transmitted miRNA, and the observed clinical characteristics in transplant recipients, remains a distant goal.

Taken together, these findings suggest that miR-218-5p expression in KP-EV as well as the capacity of KP-EV to regulate Th17/Treg ratios may be implicated in processes of graft dysfunction. Targeting these EV or miRNA represent an attractive approach for ex-vivo organ manipulation that may improve transplant outcomes.

## Data Availability Statement

The datasets presented in this study can be found in online repositories. The names of the repository/repositories and accession number(s) can be found below: https://doi.org/10.6084/m9.figshare.18131108.v1, http://www.ncbi.nlm.nih.gov/bioproject/797421.

## Ethics Statement

The studies involving human participants were reviewed and approved by Research Ethics Board of the McGill University Health Centre (2018-3831). The patients/participants provided their written informed consent to participate in this study.

## Author Contributions

AR, SN, JT, and SP designed the study. AR and SN performed the experiments, collected the data, and analyzed the results. NS provided patient data. KK performed pathway analyses. AR, SN, and SP wrote the manuscript. All authors contributed to the editing of the manuscript. All authors contributed to the article and approved the submitted version.

## Funding

This work was supported by McGill University Health Centre Foundation (Royal Victoria Hospital). This project was performed with the support of Transplant Québec, responsible for organ recovery coordination services and consent from donor families.

## Conflict of Interest

The authors declare that the research was conducted in the absence of any commercial or financial relationships that could be construed as a potential conflict of interest.

## Publisher’s Note

All claims expressed in this article are solely those of the authors and do not necessarily represent those of their affiliated organizations, or those of the publisher, the editors and the reviewers. Any product that may be evaluated in this article, or claim that may be made by its manufacturer, is not guaranteed or endorsed by the publisher.

## Supplementary Material

Supplementary Figure 1 Representative plots for Cytoflex small particle flow cytometry. **(A)** CTDR+ KP-EV were gated, followed by gating for CD9-PE, CD63-PE, HLA-DQ-PE, HLA-A2-PE and HLA-DR. **(B)** HLA-A2-PE expression in KP-EV of an HLA-A2 negative donor and HLA-A2 positive donor.

Supplementary Figure 2 Expression of miR-151-b in KP-EV correlations with recipient eGFR. **(A–F)** Correlation of KP-EV miR-151-b expression levels as measured by miRNA sequencing with respective recipient eGFR at **(A)** day 7 **(B)** day 14 **(C)** day 30 **(D)** day 90 **(E)** day 180 and **(F)** day 360 following transplantation.

Supplementary Figure 3 Expression of miR-675-5p in KP-EV correlations with recipient eGFR. **(A–F)** Correlation of KP-EV miR-675-5p expression levels as measured by miRNA sequencing with respective recipient eGFR at **(A)** day 7 **(B)** day 14 **(C)** day 30 **(D)** day 90 **(E)** day 180 and **(F)** day 360 following transplantation.

Supplementary Figure 4 Expression of miR-218-5p in KP-EV correlations with donor parameters. **(A–D)** Correlation of KP-EV miR-218-5p expression levels as measured by miRNA sequencing with **(A)** cold ischemic time **(B)** pump time **(C)** donor eGFR and **(D)** donor age.
