# Supplementary figures and images for: Extracellular Vesicles From Kidney Allografts Express miR-218-5p and Alter Th17/Treg Ratios
Source: Front Immunol. 2022 Feb 23;13:784374. doi: 10.3389/fimmu.2022.784374 (PMC8906931; doi:10.3389/fimmu.2022.784374)

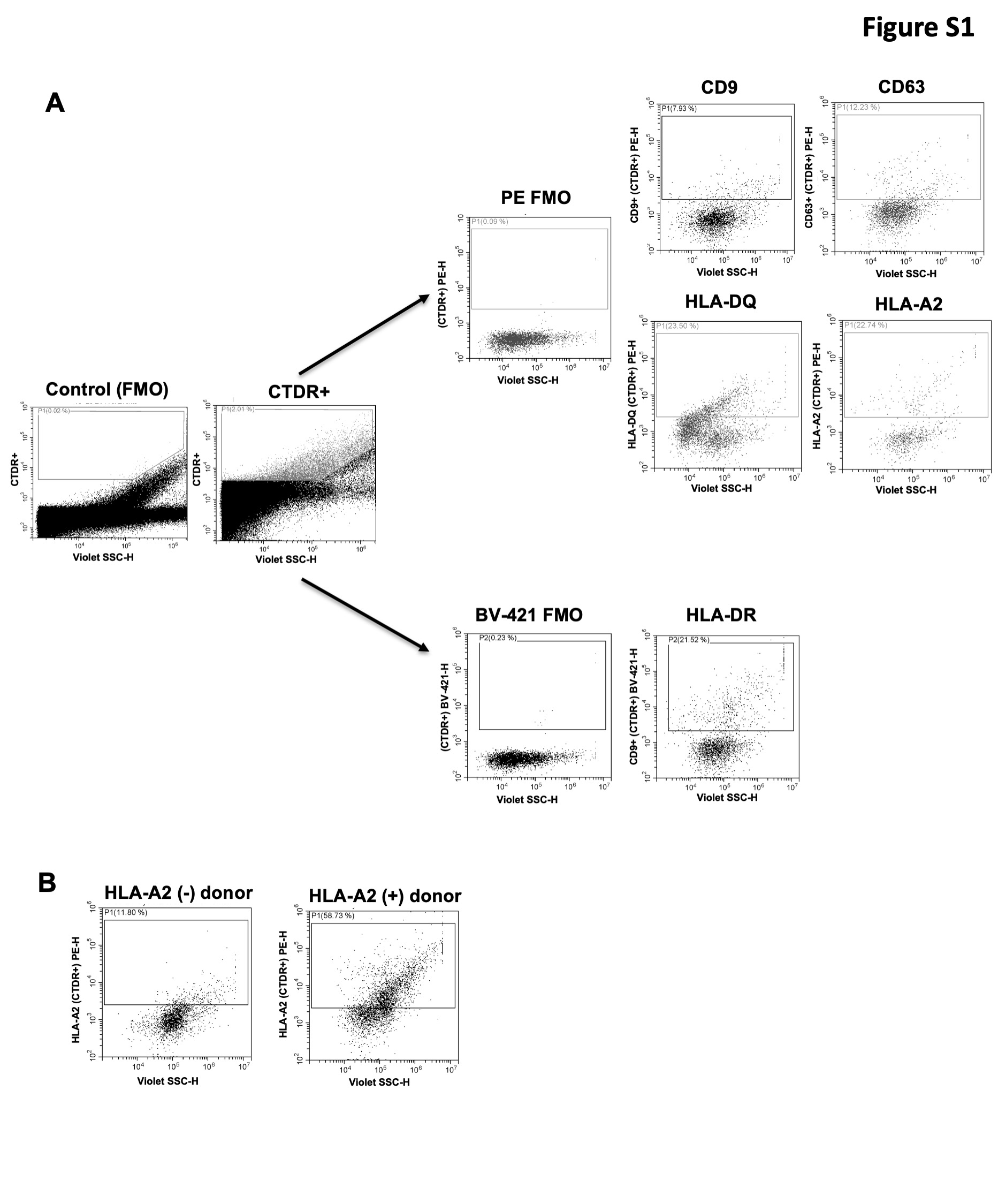

Supplement: Supplementary Figure 1 — Representative plots for Cytoflex small particle flow cytometry. (A) CTDR+ KP-EV were gated, followed by gating for CD9-PE, CD63-PE, HLA-DQ-PE, HLA-A2-PE and HLA-DR. (B) HLA-A2-PE expression in KP-EV of an HLA-A2 negative donor and HLA-A2 positive donor. [file Image_1.jpeg]

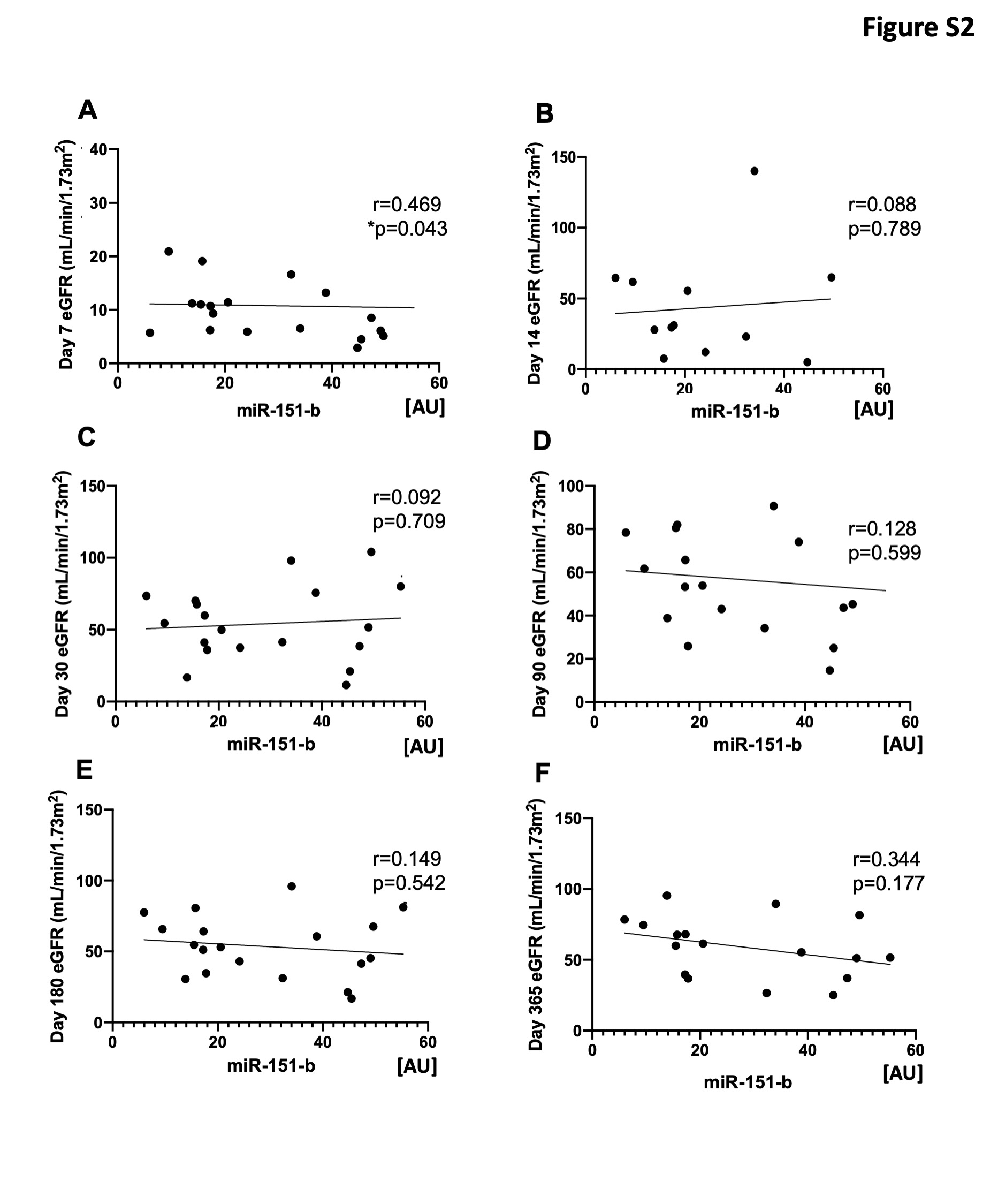

Supplement: Supplementary Figure 2 — Expression of miR-151-b in KP-EV correlations with recipient eGFR. (A–F) Correlation of KP-EV miR-151-b expression levels as measured by miRNA sequencing with respective recipient eGFR at (A) day 7 (B) day 14 (C) day 30 (D) day 90 (E) day 180 and (F) day 360 following transplantation. [file Image_2.jpeg]

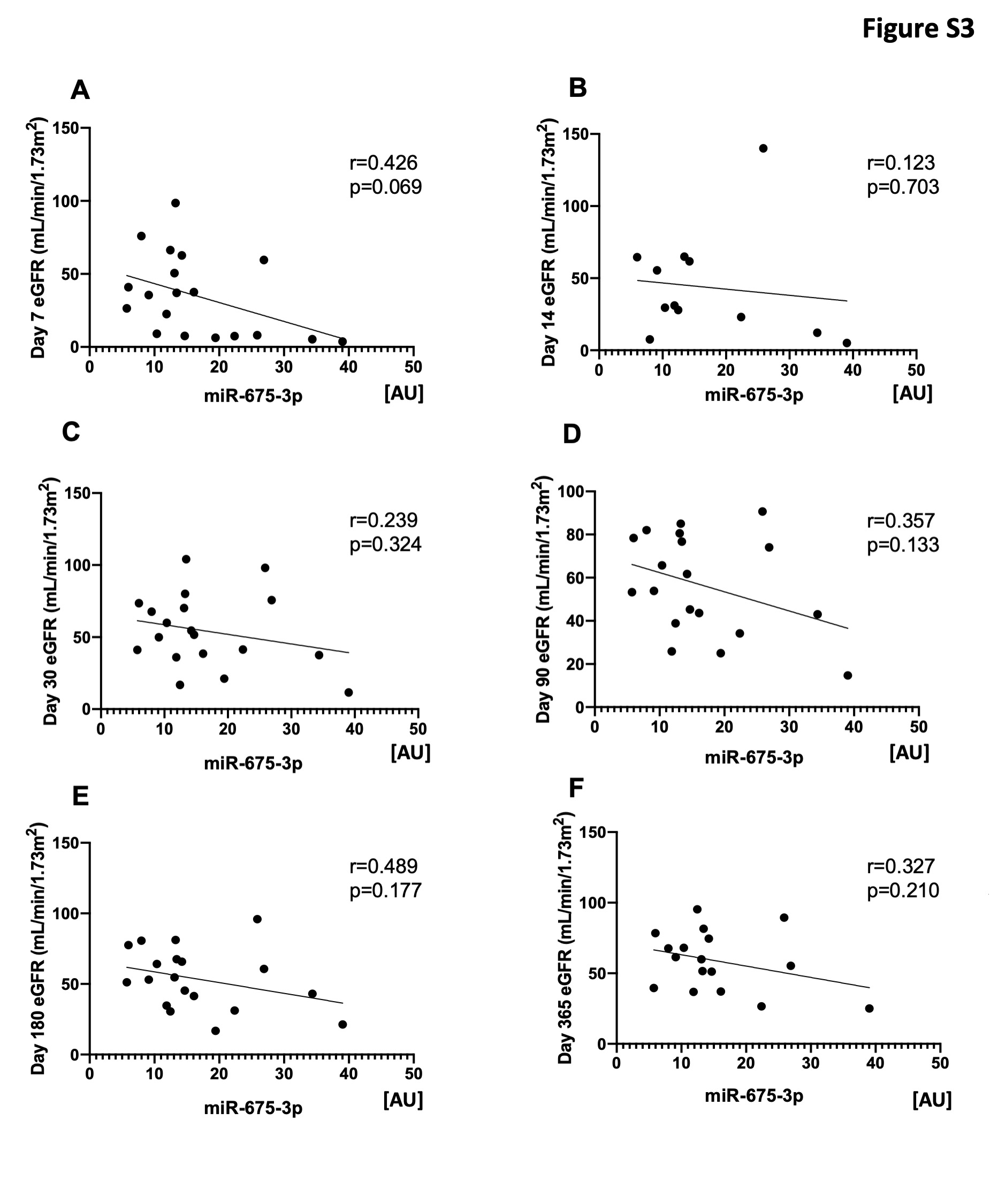

Supplement: Supplementary Figure 3 — Expression of miR-675-5p in KP-EV correlations with recipient eGFR. (A–F) Correlation of KP-EV miR-675-5p expression levels as measured by miRNA sequencing with respective recipient eGFR at (A) day 7 (B) day 14 (C) day 30 (D) day 90 (E) day 180 and (F) day 360 following transplantation. [file Image_3.jpeg]

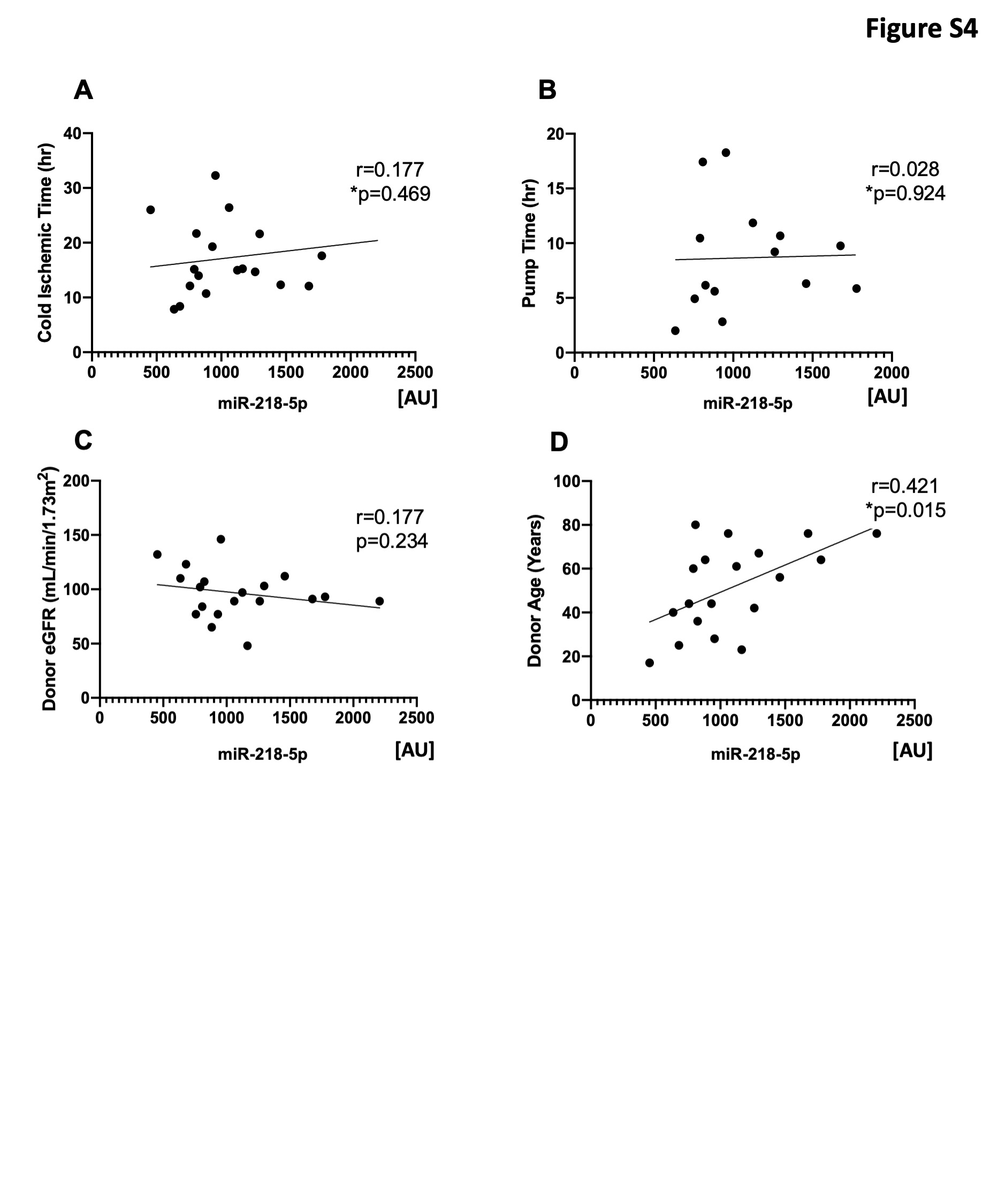

Supplement: Supplementary Figure 4 — Expression of miR-218-5p in KP-EV correlations with donor parameters. (A–D) Correlation of KP-EV miR-218-5p expression levels as measured by miRNA sequencing with (A) cold ischemic time (B) pump time (C) donor eGFR and (D) donor age. [file Image_4.jpeg]
